# The Institutional Library

**Published:** 1913-06-07

**Authors:** 


### THE INSTITUTIONAL LIBRARY.

DISEASES OF THE EYES. By C. DEVEREUX MARSHALL, F.R.C.S. Fully Illustrated. (London: University of London Press, Ltd., Hodder and Stoughton, and Henry Frowde. 1913. Price 10s. 6d.)

THE University of London Press is publishing a series of very practical text-books, of which this volume on the diseases of the eyes is a fair sample. It is designed for the use of students and those practitioners who do a certain amount of eye work, but who cannot be considered as experts. This object has been on the whole successfully carried out by Mr. Marshall. Some may object to the dogmatic teaching in places, but we entirely agree with the author that it is much more useful to describe well-tried methods than to confuse the student by giving several alternatives. Of the usefulness of this book there can be no question; the descriptions are clear and to the point; the illustrations are sufficiently numerous for the purpose of the book. The chapter on colour-blindness is an example of terse and explicit writing on a subject upon which there still exists a lamentable amount of obscurity. The theory of Dr. Edridge-Green is, of course, the one which is here so concisely explained. A useful appendix is the one giving the visual requirements of the various public services.

THE DISEASES OF THE SKIN. By WILLMOTT EVANS, M.D., F.R.C.S. With thirty-two Illustrations. (London: University of London Press, Hodder and Stoughton, and Henry Frowde. 1913. Price 10s. 6d.)

AMONG the already numerous text-books on the diseases of the skin, this well got-up manual in the series now being published by the University of London Press will surely find a place in the front rank. Without going too deeply into matters about which there still exist considerable differences of opinion (and there are many such in dermatology), this book gives an excellent account of the various affections of the skin, and the question of diagnosis is made a strong point. It is just the sort of volume that a student or a general practitioner will find useful, and one that he may be expected to have sufficient patience to read through. Dr. Willmott Evans has wisely devoted the first chapters of his book to the consideration of the anatomy, physiology, and the lesions of the skin, with a chapter on the general principles of treatment. The illustrations, although limited, are well reproduced from excellent photographs. This text-book of dermatology may not perhaps outshine some of the old-established favourites, yet it may be confidently recommended as a convenient and sufficient manual for the class for which it was written. But why not drop "anthrax" as a synonym for carbuncle?

MINOR SURGERY. By LEONARD A. BIDWELL, F.R.C.S. Second Edition. Revised and Enlarged. Illustrated. (London: University Press, Hodder and Stoughton, and Henry Frowde. Pp. 299. Price 10s. 6d.)

THE appearance of a second edition of this book is an event which it was safe to predict; we can now only congratulate the publishers on its having been called for so soon. This volume having been already noticed in our columns on its first appearance, there remains but little to add to the favourable opinion which it called forth on all sides. The death of the author as this edition was passing through the press has necessitated the final revision of the proofs being entrusted to Mr. Percy Dunn. No very important alterations are to be found, but, on the other hand, much new matter has been included, notably a chapter on bandaging and certain injuries (Chapter X.). This book appeals strongly to the general practitioner—in fact, it is just the thing that he wants. It is concise, yet detailed, and gives the most satisfactory methods of dealing with minor operations, etc., under the circumstances of general practice.

THE BOOK OF DIET. By CHALMERS WATSON, M.D., F.R.C.P.E. (London and Edinburgh: Thomas Nelson and Sons. Pp. 447. Price 2s. net.)

BOOKS on the subject of food and how it should be prepared and when it should be taken seem to be more numerous at the present time than ever. It may be suggested that the public needs as much instruction on what should not be eaten as on the variety of methods in which every conceivable edible may be prepared. Dr. Chalmers Watson has prepared a popular primer on food in general and diets in particular. The book, in the first place, is written by an acknowledged expert; secondly, it treats of the subject very fully and in popular language; thirdly, it is published at an unusually moderate price considering its bulk and range. It is more than a book of diet—it is almost an encyclopædia on foodstuffs. The valetudinarian who seeks in vain for knowledge or direction in this volume must indeed be beyond praying for. From banquets to one-course dinners, from physiology to dietetic treatment, and from breast-feeding to nonagenarian suppers, everything that civilised man puts into his mouth as food or drink is here commented upon in a practical and illustrative manner.

THE INDIAN MANUAL OF FIRST AID. By Major R. J. BLACKHAM, R.A.M.C. (Calcutta: Thacker and Spink. Price 1s.)

THIS official handbook will be, we are told, adopted as an official text-book for first-aid classes in India by the Indian Branch of the St. John Ambulance Association. It differs very little from the familiar British text-book, and it is not easy to see why the latter should not serve the required purpose equally well. If, however, some valid reason for a separate Indian manual exists, the close adherence to the original model at least ensures a satisfactory product, and Major Blackham's publication is certainly entitled to that designation.

DENTAL ANÆSTHETICS. A Text-book for Students and Practitioners. By W. E. ALDERSON, M.D. Second Edition. (Bristol: J. Wright and Sons, Ltd. 1912. Price 2s.)

THIS sound little text-book has quickly reached a second edition, which is not to be wondered at, seeing how concise and economical a manual it is. Dentists have not been slow to show their appreciation of its practical teaching. The coming generation, it is hoped, will regard anæsthetics even more seriously than their predecessors, and it will not be Dr. Alderson's fault if chloroform is not relegated to the background. Some of the statements in this book may appear to err on the side of excessive caution, but when it is realised under what conditions anæsthetics for dental operations are often administered one can only wish that the methods and warnings in this book will be more generally observed. There is little change in this edition from the first one, which was favourably noticed in our columns, and Mr. J. Bolam's description of local analgesia remains a most useful part of the book. Some new paragraphs have been added dealing with the continuous administration of nitrous oxide.

